# Mandevillian vices

**DOI:** 10.1007/s11229-024-04676-y

**Published:** 2024-07-08

**Authors:** Mandi Astola, Steven Bland, Mark Alfano

**Affiliations:** 1 Section Ethics & Philosophy of Technology, Department of Values, Technology and Innovation, Delft University of Technology, Building 31, Jaffalaan 5, 2628BX Delft, The Netherlands; 2 Department of Philosophy, Huron University College at University of Western Ontario, 1349 Western Road, London, ON N6G 1H3 Canada; 3 Department of Philosophy, Macquarie University, Levels 6 and 7, 25B Wally’s Walk, Sydney, NSW 2109 Australia

**Keywords:** Virtue, Vice, Mandeville, Cooperation

## Abstract

Bernard Mandeville argued that traits that have traditionally been seen as detrimental or reprehensible, such as greed, ambition, vanity, and the willingness to deceive, can produce significant social goods. He went so far as to suggest that a society composed of individuals who embody these vices would, under certain constraints, be better off than one composed only of those who embody the virtues of self-restraint. In the twentieth century, Mandeville’s insights were taken up in economics by John Maynard Keynes, among others. More recently, philosophers have drawn analogies to Mandeville’s ideas in the domains of epistemology and morality, arguing that traits that are typically understood as epistemic or moral vices (e.g. closed-mindedness, vindictiveness) can lead to beneficial outcomes for the groups in which individuals cooperate, deliberate, and decide, for instance by propitiously dividing the cognitive labor involved in critical inquiry and introducing transient diversity. We argue that mandevillian virtues have a negative counterpart, mandevillian vices, which are traits that are beneficial to or admirable in their individual possessor, but are or can be systematically detrimental to the group to which that individual belongs. Whilst virtue ethics and epistemology prescribe character traits that are good for every moral and epistemic agent, and ideally across all situations, mandevillian virtues show that group dynamics can complicate this picture. In this paper, we provide a unifying explanation of the main mechanism responsible for mandevillian traits in general and motivate the case for the opposite of mandevillian virtues, namely mandevillian vices.

## Introduction

In *The Fable of the Bees: or Private Vices, Publick Benefits*, Bernard Mandeville (1714/1989) argued that traits that have traditionally been seen as detrimental or reprehensible, such as greed, ambition, vanity, and the willingness to deceive, can produce significant social goods. He went so far as to suggest that a society composed of individuals who embody these vices would, under certain constraints, be better off than one composed only of those who embody the virtues of self-restraint. Although his writings caused a scandal at the time, Mandeville’s insights influenced Enlightenment thinkers such as Frances Hutcheson, David Hume, and Adam Smith (Kerkhof, 1995; Welchman, 2007). In the twentieth century, Mandeville’s insights were taken up in economics by John Maynard Keynes (1936), who coined the phrase “paradox of thrift” to refer to situations in which decision-making strategies that are beneficial at the level of the individual or household lead, in the aggregate, to economic stagnation. Smith and Keynes also associated Mandeville’s insights with the benefits of the division of labor, including specialization and cooperative action in what are now modeled as non-zero-sum games such as the stag hunt and hawk-dove interactions. More recently, philosophers such as Smart (2018a, 2018b), Alfano (2020), Astola (2021), and Bland (2022, 2024) have drawn analogies to Mandeville’s ideas in the domains of epistemology and morality, arguing that traits that are typically understood as epistemic or moral vices (e.g., closed-mindedness, vindictiveness) can lead to beneficial outcomes for the groups in which individuals cooperate, deliberate, and decide, for instance by propitiously dividing the cognitive labor involved in critical inquiry and introducing transient diversity (Smaldino et al., 2023; Zollman, 2010). These arguments have been formulated independently from, but are harmonious with, the anti-individualism that is prominent in feminist epistemology (Antony, 1995; Longino, 2022; Potter, 2006).

Importantly, for Mandeville, the public benefits of the beehive can only be reaped from individual vices when the power of individuals is “circumscrib’d by Laws.” Likewise, for Smith, the invisible hand of the market operates effectively only when the market is well regulated. Without such constraints, individual vice really does threaten to lead to kleptocracy and gangland assassinations that are inimical to prosperity. In the same vein, epistemologists who argue for the value of vice and bias similarly hold that individual epistemic vices lead to beneficial epistemic outcomes for groups only when those groups are organized in the right way. For example, Astola (2021) introduces the idea of vicious roles, such as a devil’s advocate, which only need to be filled by one or a few members of a group. However, vicious roles are not necessary for mandevillian virtues to arise. Mandevillian virtues are detrimental to or reprehensible in the individual possessor, but they are systematically beneficial to the group to which that individual belongs. We suggest that mandevillian virtues have a negative counterpart, mandevillian vices, which are traits that are beneficial to or admirable in their individual possessor, but are or can be systematically detrimental to the group to which that individual belongs.

Whilst virtue ethics and epistemology prescribe character traits that are good for every moral and epistemic agent, and ideally across all situations, mandevillian virtues show that group dynamics can complicate this picture. In this paper, we aim to do two things: provide a unifying explanation of the main mechanism responsible for mandevillian traits in general, and motivate the case for the opposite of mandevillian virtues, namely mandevillian vices. In Sect. 2, we argue that mandevillian effects are the result of dispositional diversity, which explains why the manifestation of vice is beneficial in some cases. In Sect. 3, we show that virtues such as mercy and forgiveness along with their epistemic counterparts, can become mandevillian vices when everyone possesses them. In Sects. 4 and 5, we argue for cases of epistemic mandevillian vice whilst explaining the role of dispositional diversity in causing them: Sect. 4 focuses on apt deference and Sect. 5 on open-mindedness. In Sect. 5, we also address a possible counterargument to dispositional diversity being the most important cause of these mandevillian effects. The counterargument is that uniform, not diverse, levels of myside bias have been shown to be beneficial for group cognition. While this might be the case, we argue that collective deliberation is often structured in ways that yield diverse levels of myside bias precisely because this amplifies its positive effects. We argue that even in those cases, having a non-biased moderator, who is uniquely unbiased, typically harnesses the myside bias of others to produce the positive effects. In Sect. 6, we address a problem with the division of labor model of epistemic and moral virtue: the fact that such moral and epistemic divisions of labor are often created by unfair norms associated with gender, race, and other social categories. We conclude in Sect. 7 with some implications. Instead of trying to homogenize people to a procrustean ideal of moral or epistemic virtue, it may be better to harness the diversity and variance of traits in groups via incentives and role assignments in pursuit of valuable moral and epistemic ends.

## Mandevillian vices and dispositional diversity

Before addressing mandevillian vices, it will be helpful to clarify what philosophers mean by mandevillian virtues. Smart (2018a) coined the term ‘mandevillian intelligence’ to denote “a specific form of collective intelligence in which individual cognitive vices are seen to play a positive functional role in yielding collective forms of cognitive success.” For example, Mercier and Sperber (2011, 2017) argue that myside bias and intellectual laziness lead to an efficient division of cognitive labor in interpersonal argumentation. When interlocutors are biased against different views, they often vet those views more thoroughly than an open-minded individual typically would through solitary reflection. Astola (2021) has extended this notion to the moral realm, where she characterizes mandevillian virtues as “character traits that are typically seen as moral vices at the individual level [but which] play a structural role in constituting this ethical behavior at the group-agent level.” She points out that vindictive individuals are more likely than virtuous agents to punish norm transgressors, which can help ensure members’ safety and uphold important moral norms (Forber & Smead, 2014). More generally, we refer to mandevillian virtues as personal vices that have beneficial effects at the level of collectives.

If the manifestation of vicious behavior can be beneficial in collective contexts, it stands to reason that strictly virtuous behavior can be too much of a good thing in those same contexts. A similar view is defended in the philosophy of science by Mayo-Wilson et al. (2011) under the name of the *independence thesis*: rational individuals can form irrational groups, and rational groups can be made up of irrational individuals. We aim to extend this view to virtue theories by arguing that there are *mandevillian vices*, which are the counterparts of mandevillian virtues. Mandevillian vices are *dispositions that are typically seen as strengths or admirable traits at the individual level, but which can play a negative functional role in supporting the emergence of disvaluable behavior or outcomes at the collective level*. We argue below that open-mindedness and mercy, when manifested thoroughly and uniformly, are two such vices.

The normative status of mandevillian traits depends on their epistemic and moral consequences at the level of collectives, or what we call their *mandevillian effects*. They are what Battaly (2018) calls *effects-virtues and -vices*. Battaly contrasts effects-virtues and -vices with *responsibilist* traits whose normative status depends on their constitutive motivations and values. It should be noted, however, that for many responsibilists, the status of traits doesn’t depend exclusively on their motivations and values; effects matter too. Virtues must be conducive to valuable epistemic or moral ends. For example, Zagzebski claims that epistemic virtues must facilitate “cognitive contact with reality” (Zagzebski, 1996, 167); their doing so is a necessary but insufficient condition of their status as virtues. For these responsibilists, a trait’s playing a positive or negative role in collective cognition, by itself, does not qualify it as a virtue or vice. While we don’t want to exclude the possibility that mandevillian traits could be virtues or vices in the responsibilist sense as well, we will not argue for that position here. Rather, we will treat their normative status as being dependent on their epistemic and moral effects in collective contexts.

Our account is inspired by Page’s diversity-trumps-ability theorem (Page, 2007). Page recognizes that both cognitive diversity (e.g., diverse perspectives, heuristics, interpretations, and mental models) and cognitive ability (e.g,. intelligence, competence) are important to collective problem-solving. But there is a trade-off between them because agents who are high in cognitive ability tend to think similarly. According to Page, when large groups are faced with difficult problems, they are more likely to succeed when membership is determined randomly rather than selected on the basis of cognitive ability (Ibid. 10). Randomly selected groups are more likely to be cognitively diverse, and diverse groups generate more strategies, solutions, and improvements than cognitively homogenous groups, even when those groups are made up of talented individuals. This is particularly important when groups face complex problems, where many potential solutions must be tested in order to find the one that works best. The same insight underlies Fishkin’s (2003) approach to deliberative polling, which we discuss in more detail below. Research in foraging theory suggests that this result holds for both human and non-human animals (Aljadeff et al., 2020; Sun et al., 2013).

Cognitive ability is an almost unalloyed epistemic good *for individuals*: greater ability leads to better beliefs, predictions, and solutions.^1^ For *collectives*, by contrast, cognitive ability has diminishing returns: increasing cognitive ability decreases cognitive diversity, and the latter is more valuable than the former when a group is faced with difficult problems. Diversity also means that the risk profiles of group members differ, leading some to be more risk-seeking while others are more risk-averse. This can be beneficial in multiple ways: the risk-seekers explore unpromising terrain that others avoid, potentially unlocking benefits for the group, but at the same time if they fail catastrophically the group may still survive due to the presence of risk-averse members (Bicchieri, 2016, Ch. 5).

We contend that a similar dynamic, harnessing dispositional diversity, is responsible for mandevillian effects. By ‘dispositional diversity’, we mean a diversity of moral and intellectual traits, competences, and capacities, though we focus exclusively on traits. Aristotle (1934) observes that “goodness is simple, badness manifold” (*NE*, 1106b, 35). This is explained by his doctrine that virtues are states of character that lie between vices of excess and deficiency. For example, the virtue of courage occupies the mean between the vices of recklessness and cowardice. Intellectual virtues can also be, and have been, conceptualized as conforming to the doctrine of the mean (King, 2021, 26). Intellectual humility, for instance, arguably occupies the mean between the vices of intellectual arrogance and servility.

For Aristotle, the doctrine of the mean explains the rarity of virtue: since there are more vicious states than virtuous ones, and there is no reason to think that people are born virtuous, it is difficult to consistently manifest the virtues without exerting considerable effort and engaging in careful reflection (*NE*, 1109a). More importantly, for our purposes, it implies a *virtue-diversity trade-off* in collectives: the more virtuous agents are in a particular respect, the more they will resemble one another in that respect. Courageous people are alike with respect to their courage, but those who lack the virtue of courage may be cowardly or reckless, and to different extents. The virtuous are good in one way; the vicious are bad in many. And, as is the case with cognitive ability, we argue that diversity—*dispositional* diversity—has overlooked benefits in collective contexts that can trump the moral and epistemic goods achieved by strictly virtuous behavior. Even Smaldino et al. (2023), who catalogue multiple types of diversity, do not address dispositional diversity of the sort we consider here.

These benefits, unlike the benefits of cognitive diversity, are not reliably achieved through random sampling. Random sampling is beneficial when collectives are faced with difficult cognitive problems because no one knows ahead of time which way of thinking will yield positive results. Thus, cognitive diversity increases the chances of collective success, regardless of how agents differ. By contrast, dispositional diversity is valuable insofar as it is necessary for achieving the equilibria that lead to collective flourishing. One such equilibrium is what Kuhn (1977) calls ‘the essential tension’ between intellectual independence and servile conformity that enables scientific progress. Another is the balance between open-mindedness and closed-mindedness that yields an efficient division of cognitive labor in collective deliberation. A third is the mixture of vindictiveness (and resultant second-party and third-party punishment) and forgiveness that leads to cooperation. These equilibria cannot be achieved in contexts where everyone adopts the same strategy, as would be the case in collectives of uniformly virtuous individuals. Mandevillian traits are effects-virtues to the extent that they promote a valuable equilibrium within a particular collective, and effects-vices to the extent that they interfere with the realization of such an equilibrium. Like other effects-virtues and -vices, their normative status is context-dependent, and specifically dependent on features of the social contexts in which they are manifested (Astola, 2021; Bland, 2024).

It’s worth noting that this is a departure from Mandeville, who claims that *uniformly* distributed vices, such as selfishness and acquisitiveness, yield positive effects in open markets (when constrained by benevolent laws). In his view, we are better off, in the aggregate, when *everyone* is selfish and acquisitive. Our view is that this is rarely the case. Rather, positive outcomes at the collective level are facilitated by propitious forms of dispositional diversity, which can be increased through the limited manifestation of traditional vices. Consider another classic example of market behavior: markets and their participants are well-served by including a critical mass of entrepreneurs who have an appetite for extreme risk-taking (Knight, 1921). We encourage this behavior, even though it can be irrational from the individual’s point of view, by reducing their risk of personal ruin; this is one rationale for laws of incorporation and limited liability. On the other hand, too much risk-taking would lead to collective ruin; we can encourage such behavior only because we know it is relatively rare and counter-balanced by overwhelmingly safe investments. Wealthy economies must strike this balance, and they would struggle to do so without dispositional diversity. The same is true, we argue, of collective moral and intellectual flourishing.

## Mercy and forgiveness

Morality is, to a large extent if not entirely, about cooperation. By cooperation we understand evolutionarily stable solutions to non-zero-sum games that are recurrent in our lives. To date, the most well-developed theory of morality-as-cooperation is due to Curry (2016; see also Curry et al., 2021), who posits seven distinct types of cooperation grounded in evolutionary game theory. These are kin altruism, reciprocity, group solidarity, hawkishness, dovishness, fair distribution of resources and labor, and respect for prior ownership. Let us consider a well-studied type of cooperation: reciprocity in iterated prisoner’s dilemmas. One relatively successful strategy in the iterated prisoner’s dilemma is the copycat or tit-for-tat strategy (see Axelrod, 1984, among many others). A player following this strategy initially cooperates, and then either cooperates or defects in accordance with whatever their partner did in the previous round. This strategy, while stable, can lead to suboptimal interactions in cases where the other player accidentally defects or suffers from a bout of weakness of will. In such cases, a cycle of negative reciprocity ensues.

A more “forgiving” strategy is the copykitten or tit-for-two-tats strategy (Axelrod, 1984).^2^ A player following this strategy initially cooperates, and then continues to cooperate unless their partner defects twice in a row. The copykitten also reverts to cooperating if their partner acts cooperatively once, even after multiple defections.^3^ The copykitten strategy—and its underlying virtue of forgiveness—is stable and beneficial in many circumstances, but not all (Axelrod, 1984). When there are too many aggressive potential partners, the copykitten is easily exploited, making a collection of copykittens vulnerable to invasion by agents who adopt more pugnacious strategies. Likewise, when the communicative environment is so noisy that what seem like agreements to cooperate are often not mutually understood, the copykitten strategy is easily exploited. And in an evolutionary game-theoretical context, people will abandon the strategies and dispositions that systematically fail them and their peers. In other words, copykittens who embody the virtue of forgiveness are great to have around until there are too many of them, at which point the group becomes vulnerable to exploitation. It’s also worth bearing in mind that the effectiveness and vulnerability of the copykitten strategy depend on the social constraints that actors face. If partner choice and third-party sanctioning are introduced, the copykitten strategy again becomes viable (Fehr & Fischbacher, 2004; Martin & Cushman, 2015). This is because the copykitten can choose to switch to a more cooperative partner after being exploited multiple times by players employing an aggressive, defection-heavy strategy. And the copykittens can also be protected by one or a few vindictive members of the group. The mandevillian virtue of vindictiveness may, in this way, forestall the bad effects of the mandevillian vice of forgiveness. This insight suggests that collectives benefit from a moral division of labor between forgiving individuals and vindictive ones. When copykittens are counterbalanced with reliablethird-party punishers, the group may become more cooperative and resilient.

The idea of a moral division of labor is useful here: what is morally right for a person to do depends on their role and relation to others. This idea has been used in moral philosophy to explain the apparent contradiction in personal and civic duties. If there is one set of duties that clearly conflicts with another, then a possible explanation is that those duties need to be fulfilled by different agents (Scheffler & Munoz-Dardé, 2005). That being said, we are also keenly aware that disadvantaged members of a society (e.g., women, people of color) are more likely to be assigned forgiving roles, which can simultaneously enhance overall group wellbeing and lead to increased relative disadvantage (Cherry, 2023; O’Connor, 2019). We return to this point in Sect. 5.

Another virtue that exemplifies the upside of the division of moral labor is mercy. Being merciful is typically seen as a moral virtue. Mercy can be seen as the prerogative of the sovereign (Locke, 2011, 755). One can exercise mercy only when one has power. When too many people exercise mercy, or enough of them exercise it too bountifully, negative social outcomes are likely. Consider the case of a police officer letting someone off with a warning. In many cases, letting someone off without a fine might be justified on the basis of mercy. Imagine a police officer during a routine check encountering a stressed parent dropping his kids at school, tears almost welling up in his eyes when he realizes he has forgotten his driver’s license. If every traffic cop were to exercise mercy in this way, the result could be a shift in norms that leads to poorer road safety. A little mercy goes a long way, but too much could lead to large-scale harm. Mercy, like forgiveness, must be non-universal to be virtuous.

If these reflections on mercy and forgiveness are on the right track, we face the following paradox: at the moment that too many people possess them, they become mandevillian vices (Benbaji & Heyd, 2001; Williams, 1996). This may be why these virtues have often been described as supererogatory (Benbaji & Heyd, 2001). Their supererogation points to the fact that one is permitted not to be merciful or forgiving in many cases, and this is because the lack of mercy or forgiveness serves the function of justly punishing norm transgressors, and deterring others who might otherwise transgress.

Virtues with this character are prone to being mandevillian vices. And this is explained by a need for dispositional diversity. Dispositional diversity creates an efficient division of labor through which the lenience of one subgroup is kept in check by the stringency of another subgroup. Moral systems require both norms of punishment and norms of leniency. We contend that it is often more efficient to divide the labor of upholding and enforcing both kinds of norms among different people, rather than expecting every member of the group to embody the golden mean individually. The people who are good at punishing others might be less likely to be the ones who uphold norms of lenience. The wise king Solomon meting out both justice and mercy may be possible in theory, but in practice it is often better to divide these roles among people with different tendencies and dispositions. If this is right, dividing the labor of norm enforcement can be more efficient than expecting everyone to be virtuous. Dispositional diversity means that different people are likely to fill different roles effectively.

Mercy and forgiveness also have epistemic counterparts with a similar saturation-point structure. For instance, epistemic toleration describes a non-punishing attitude towards others with viewpoints that are seen as bad in some way. Epistemic toleration also includes the conviction that not punishing or excluding others has important benefits that are worth preserving (Straßer et al., 2014). A lack of intolerance may cause a weakening of scientific norms, which might be epistemically disadvantageous. Other epistemic virtues, such as apt deference and open-mindedness, also have this feature, as we argue below.

## Social learning and apt deference

Philosophers have traditionally overestimated the role of individual learning in our epistemic lives. We are now coming to the realization that most of our knowledge is social; indeed, it may even be irreducibly social (Green, 2017; Levy, 2022; Levy & Alfano, 2019). Our most fruitful source of knowledge about the world is not the world itself, but other human beings. Acquiring this knowledge, however, requires manifesting the virtue of *apt deference* (Ahlstrom-Vij, 2019). Like other virtues, apt deference can be understood as occupying the mean between the vices of incredulity and gullibility (Robertson, 2016). Agents who defer infrequently miss out on massive epistemic goods and opportunities; agents who defer indiscriminately end up being misled. And those who claim to be thinking for themselves and doing their own research may end up deferring without realizing it, often to untrustworthy sources (Meyer et al., 2021).

Humans are adept social learners who use a variety of heuristics to identify trustworthy sources of testimonial knowledge (Henrich, 2016). Among them is the tendency to conform to the majority (Muthukrishna et al., 2016). This strategy is especially adaptive in conditions where the Condorcet Jury Theorem holds. The Condorcet Jury Theorem establishes that aggregate predictions outperform the predictions of individual forecasters when groups are large, competent, and independent. Indeed, when these groups are sufficiently large, they approach perfect accuracy. To the extent that agents are aware of this, it is rational for them to automatically defer to the wisdom of crowds, rather than exercising their own judgment. And it seems that agents actually are aptly deferential to the wisdom of crowds in many cases (Mercier & Morin 2019). However, widespread deference compromises the independence on which the wisdom of crowds depends; as deference increases, the accuracy of the crowd comes to resemble the accuracy of an individual instead of surpassing it. According to Page’s diversity prediction theorem, prediction diversity is as important to collective accuracy as individual ability (Page, 2007, 208). Thus, following the crowd makes the crowd less wise, but ignoring the crowd makes individuals less wise. We call this the *Condorcet conundrum*.

De Courson et al. (2021) claim that crowds optimize both collective and individual accuracy when they include a mix of deferential conformists and independent non-conformists. They argue that we have evolved to approximate this “virtuous equilibrium” because of our taste for originality, i.e., our preference, all things being equal, for divergent beliefs and opinions (Mercier & Morin 2019).^4^ This preference creates an incentive for status-seeking individuals to manifest intellectual independence that can outweigh the epistemic perils of deviating from the crowd. Their modeling shows that a distribution of individuals who are variously concerned with social influence and originality—i.e., dispositional diversity—yields a mutually beneficial division of labor.

De Courson et al. (2021) model several Curty-Marsili games in which agents make binary forecasts, either by relying on their own information or by deferring to others. They embed these agents in an evolutionary network where learning behavior is determined by two genes: a strategy gene with follower alleles and information-seeking alleles, and an originality gene that determines taste for originality (either 0 or > 0). These genes yield 3 phenotypes: followers who prefer originality (non-conformists), followers with no preference for originality (conformists), and information seekers for whom the originality gene makes no difference (independents). Fitness is determined by forecasting performance, information gathering costs incurred, and audience share. Low fitness agents are selected against and replaced with clones of ‘living’ agents. The odds of any given genetic combination being a replacement is proportional to its fitness at the time of replacement.

The payoff of an agent’s audience share depends on the incentive to maximize followers. De Courson and colleagues found that when the incentive is too weak, information seekers go extinct, since they are less accurate than followers and accuracy is valued more than popularity. Without the information they gather, viewpoint diversity collapses and the wisdom of the crowd is lost. When the incentive is too strong, non-conformists do not spread; because they decouple their beliefs from the beliefs of the crowd, their scores are more widely dispersed, which results in them having fewer followers on average. And since independent information seekers depend on non-conformists, they too disappear, leaving only poorly informed conformists.

However, de Courson and colleagues find that, at low to moderate levels, the popularity motive yields a stable minority of information seekers that co-exists with larger groups of followers, both conformist and non-conformist. This mixture yields a propitious division of cognitive labor: individual learners collect reliable information without appealing to others, non-conformists sustain viewpoint diversity, and conformists harness the wisdom of the crowd. Consequently, both viewpoint diversity and global accuracy are fostered.

Crucially, this division of labor would be unstable if agents cared only about accuracy: both information seekers and non-conformists would switch strategies, in favor of conformity, to benefit from the wisdom of the crowd. This apt deference is entirely rational, and intellectually virtuous, from the perspective of each individual. However, it will quickly destroy the wisdom it seeks to exploit. For this reason, it is a mandevillian vice. Though social learning is generally superior to individual learning, the former cannot exist in the absence of the latter.

As mentioned above, Kuhn describes a similar equilibrium as being essential to scientific progress; he calls it “the essential tension” (Kuhn, 1977). On his view, science benefits from long periods of relative stasis—normal science—punctuated by sudden episodes that culminate in the replacement of prevailing paradigms—revolutionary science. Normal science is a puzzle-solving activity whose problem space is defined by the theoretical frameworks that scientists inherit via an extreme deference to previous generations through textbook learning, lab lore, equipment that cannot be easily or cheaply replaced, and analytic and statistical methods that most can use but do not fully understand. Physicists in the nineteenth century learned how to see and solve problems in a Newtonian world by uncritically imitating their teachers. Revolutionary science occurs when rogue scientists propose an entirely new theoretical framework in response to a disciplinary crisis. Einstein’s theories of relativity solved the anomalies that beset Newtonian physics by introducing a novel conceptualization of space–time. We know the names of scientific revolutionaries such as Einstein because they succeeded, but most don’t.^5^ And, Kuhn emphasizes, there’s no way for them to know ahead of time that their theories have the resources to solve the puzzles to which they will give rise; they must take it on faith. Thus, as a collective endeavor, science benefits from the intellectual independence of a handful of scientists who don’t defer to the majority, even though it would be epistemically rational as individuals for them to do so. Their number can’t be too large, however; otherwise normal science cannot make progress through the puzzle-solving that presupposes an existing paradigm. The essential tension is the virtuous equilibrium between widespread deference and occasional independence within the scientific community. While a taste for originality is one way of achieving this balance, philosophers and sociologists of science have also identified incentive structures as serving the same epistemic function (Kitcher, 1990; Merton, 1957; Strevens, 2003).

Laypeople face a similar dilemma when it comes to their consumption of media and social media, especially journalism about complex topics that require specialist knowledge, such as science, government policy, and international relations. Consider the case of an educated adult without any particular medical expertise who is deciding whether to get vaccinated against COVID. They could, if rich and powerful enough, run their own randomized controlled trial and analyze the results. Less ambitiously, they could read widely and form their own opinion about the peer-reviewed papers that purport to establish the safety and effectiveness of a particular vaccine. Even less ambitiously, they could read a few pieces of science journalism. Less ambitiously still, they could check what their family, friends, and connections on social media are saying. Of course, in the recent pandemic, almost everyone took one of the less ambitious approaches. From the mandevillian point of view, this is not only acceptable but desirable, on the assumption that people’s social networks are structured in such a way that enables them to exploit the wisdom of crowds. Unfortunately, recent research suggests that only a small minority of them was so-positioned, at least when it came to discourse about vaccines (Klein et al., 2022; Sullivan et al., 2020). This returns us to the point, made above, that whether a disposition counts as a mandevillian virtue or vice depends on the structure and organization of the group in which its bearer operates. Apt deference is virtuous when there are independent thinkers in the collective and the group is structured to preserve independence. When independent thinkers get shouted down or independence is undermined (especially when it is undermined surreptitiously so that people continue to think that it holds—see, e.g., Benkler et al., 2018), the wisdom of crowds cannot be harnessed.

## Open-mindedness and collective deliberation

Much of the literature on mandevillian virtues has focused on myside bias, which is characteristic of closed-mindedness (Smart 2018a, 2018b; Bland, 2022, 2024). However, some of the conclusions that have been recently drawn from simulations pose a threat to our account of the main mechanism responsible for mandevillian traits. Before articulating and responding to this threat, let us briefly explain myside bias and its effects on the reasoning of individuals.

Myside bias is the common tendency to pursue and accept information that favors what we already believe and ignore or discount discordant evidence. This tendency fits Battaly’s characterization of closed-mindedness as “an unwillingness or inability to engage (seriously) with relevant intellectual options” (Battaly, 2018, 262). Myside bias leads to several epistemic shortcomings: belief perseverance (Anderson et al., 1980), forecasting inaccuracy (Haran et al., 2013), overconfidence (Koriat et al., 1980), polarization (Tesser, 1973), and the illusion of objectivity (Kunda, 1990). For these reasons, it seems that individuals are epistemically better off to the extent that they can resist myside bias and exhibit open-mindedness. On the other hand, they shouldn’t be so open-minded that, as the adage goes, their brains fall out. We should openly engage only with relevant intellectual options—information, evidence, arguments, etc.—and only to the extent that they merit our engagement. We can safely ignore the claims of the flat earth community without being closed-minded. Thus, open-mindedness occupies the virtuous mean between closed-mindedness and intellectual diffidence (Stanovich et al., 2016, 208).

While myside bias may have deleterious effects on the reasoning of individuals in isolation, Mercier and Sperber argue that it serves an important function in dialogical contexts: it efficiently distributes the cognitive labor required to thoroughly vet multiple viewpoints on the same topic (Mercier & Sperber, 2011, 2017). As long as all of the relevant views are represented within a group, every view gets thoroughly defended by its advocates and criticized by its detractors. In this way, collectives can be more thoroughly open-minded than their members, but only when myside bias is moderate. Discussants must be willing to change their minds when presented with strong evidence that they are mistaken.

This view is supported by formal modeling, which finds that strong myside bias leads to bi-polarization, but moderate myside bias facilitates faster and more accurate consensus formation than unbiased reasoning (Banisch & Shamon, 2023; Gabriel & O’Connor, 2022). In these cases, however, mysided reasoning is a mandevillian virtue *without* involving dispositional diversity. The agents in Gabriel and O’Connor’s simulations, for instance, are *homogeneously* biased. For this reason, it is an unrealistic simulation of real deliberation. Myside bias differs across individuals and even within individuals, depending on the strength of their convictions (Stanovich & West, 2008; Taber et al., 2009; Shamon et al., 2019).

To better accommodate this fact, Baccini et al. (2023) generated a Bayesian model in which myside bias is distributed heterogeneously over populations of deliberating agents. They found that group deliberation generally had a negative effect on a group’s chances of reaching an accurate consensus, except when myside bias is distributed asymmetrically across initially correct and incorrect reasoners. In these cases, both consensus and truth-tracking increased as levels of myside bias increased among correct reasoners. When those who were least likely to change their minds were most likely to be correct, the group benefited from their closed-mindedness.

This type of closed-mindedness is *not* a mandevillian virtue since it benefits individuals *independently* of how it affects the collectives to which they belong. Individuals who possess epistemic goods in a particular domain—accurate, justified beliefs; understanding; etc.—risk losing those goods when they engage with relevant intellectual alternatives. They are better off ignoring or discounting those alternatives than they are giving them a fair hearing. This is Kripke’s dogmatism paradox (Kripke, 2011). More epistemically impoverished agents, on the other hand, are well served by remaining open-minded in order to learn from others. Thus, groups benefit from dispositional diversity, but not because effects-vices at the individual level yield positive effects at the level of collectives. Closed-mindedness is an effects-vice only for the ignorant in deliberative contexts. To the extent that we are concerned with the epistemic wellbeing of the least-well-off (what might be called an *epistemic minimax* principle—see Bashardoust et al., 2023; Alfano et al. forthcoming), we should encourage the informed to be closed-minded while entreating the ignorant to be a bit more gullible.

In summary, the results from recent simulations of myside bias in collective deliberation seem to challenge our account of the mechanism responsible for mandevillian traits. They find both that mandevillian traits emerge from homogenous trait distributions and that heterogeneous trait distributions do not yield mandevillian virtues and vices. Naturally, these findings call for a response.

The problem with relying too heavily on these results, from our perspective, is that the simulations ignore the *role-dependent effects* of myside bias. This limitation is admirably recognized by Baccini et al.: “in our model the speakers are selected randomly: at each step, any agent can be picked to be the next one to present an argument. In many contexts this is not the case. For instance, hearings in a court of law or in a parliament chamber are not random, and there is a clear protocol that determines who gets to speak at which time” (Baccini et al., 2023). As they point out, adversarial deliberation works best when it’s well structured, such that different agents have different roles to play and everyone follows the same procedural protocol. In adversarial (as opposed to inquisitorial) systems of criminal law, for example, both the prosecution and the defense are expected to reason in ways that manifest a pronounced bias in their favour; in fact, they’re not doing their jobs competently otherwise. These systems also include adjudicators—judges, juries—who are supposed to be as unbiased as possible. When everyone is doing their jobs well, the unbiased adjudicators are more accurate than the biased litigators, but the accuracy of the former depends on the arguments of the latter. The legal system is designed to harness dispositional diversity to generate mandevillian virtues.^6^

Consider another form of highly structured collective deliberation: deliberative polls. Deliberative polling involves selecting a random representative sample of citizens to deliberate about an important and contentious issue facing their polis. Participants are given carefully balanced briefing materials before being randomly assigned to small discussion groups, led by trained moderators, where they develop questions for a panel of competing experts and politicians. Once the plenary Q-and-A session is complete, participants complete a confidential questionnaire whose results are broadcast to the larger public. Fishkin emphasizes that “Every aspect of the process is designed to facilitate informed and balanced discussion” (Fishkin, 2009, 26). This is not accomplished by selecting political moderates with open minds, but by aggregating people with a representative range of views. It is crucial, however, that they are sufficiently open-minded to change their minds in response to the evidence and arguments that emerge from the deliberative process. And it seems they often are. Participants complete identical questionnaires at the beginning and the end of the deliberative poll, which reveal that their beliefs and attitudes tend to shift significantly over the course of the process, though not in ways that indicate conformity or polarization (Ibid. 121). By contrast, it is important that panelists be sufficiently partisan to function as effective spokespersons for their positions; open-mindedness is a mandevillian vice when exhibited by individuals in this role. Finally, the organizers of deliberative polls must be extremely open-minded; they must provide information packages that highlight the strongest lines of evidence for every position under discussion. It is the role-based distribution of epistemic dispositions that facilitates informed and balanced discussion in deliberative polls.

Another limitation of Baccini et al.’s study is its reliance on majority rule to assess collective accuracy. In many collectivist contexts, judgements and decisions are left to a single individual—judges in courtrooms; CEOs in boardrooms; generals on battlefields; ministers and heads of state in government—who are presented with relevant information and arguments by a body of advisors and stakeholders. An efficient division of cognitive labor can be achieved if the body contains biased advocates, but likely won’t be achieved if the decision-maker is closed-minded. When leaders are noticeably biased in their contributions to collective deliberations, they tend to silence dissenters and encourage conformity (Nemeth, 2018; Sunstein & Hastie, 2015). As a result, the group ends up reasoning like a biased individual rather than leveraging the biases of its constituents. In these contexts, open-mindedness is a mandevillian vice when manifested by most discussants but not by leaders and decision-makers. Once again, it’s the balance of open-mindedness at the executive level and closed-mindedness at the consultative level that yields mandevillian traits and collective wisdom.

## Equity, justice, and the division of moral and epistemic labor

One final consideration we wish to return to concerns the justice-relevant implications of endorsing the division of both moral and cognitive labor. Talk of division of labor is likely to conjure images of the Industrial Revolution in Manchester, slave labor on American cotton plantations, and Ford Model-T factories. In turn, these images may remind us of stereotypes of obedient, docile workers and commandeering employers and slavers. While there is no doubt that such social arrangements can harness the division of labor and increase overall productivity, these sorts of economic structures also tend to produce and reinforce stark, violently-enforced differences in the distribution of benefits and burdens. Looking back further in human history, the rise of sedentary agriculture enabled specialization, divided labor, and increased overall economic output. It also led to massive increases in inequality (Henrich, 2016). Looking back even further, arguably the first and most robust division of labor in human history has been on the basis of gender or sex; indeed, there are no documented cases of stable human societies that do not have a gendered division of labor (O’Connor, 2019). Other divisions of labor rely on typing people not only by class and gender but also by race, ethnicity, caste, and religion. Plato’s myth of the metals in *Republic* is yet another example, though one that interestingly eschews gendered typing.

Our arguments about mandevillian virtues and vices might be seen to implicitly support the reinforcement or reemergence of type-based divisions of moral and epistemic labor that activists, politicians, free thinkers, and others have spent centuries underming. Suppose, for instance, that we are right that it is good, at the collective level, for some members of a group to be forgiving while others are vindictive. It would not be a huge surprise if women were more often expected to be forgiving and adopt the role of peacemakers while men were more often expected to be vindictive and adopt the role of enforcers. Do we really want to reintroduce the idea of feminine and masculine virtues, as some conservatives have proposed (Mansfield, 2006)? Suppose that we are right that it is good, at the collective level, for some members of a group to be status-seeking intellectual mavericks while others are deferential. It would not be a huge surprise if Brahmins were more often expected to be brilliant thinkers and adopt the maverick role while Shudras were more often expected to defer to consensus or to a specific thought-leader. Do we really want to reinforce caste-based stereotypes and expectations that could shape people’s lives, prospects, and self-conceptions?

Nietzsche (2001) discusses this phenomenon in *The Gay Science* 21, saying:

A man’s virtues are called *good* depending on their probable consequences not for him but for us and society: the praise of virtues has always been far from “selfless,” far from “unegoistic.” Otherwise one would have had to notice that virtues (like industriousness, obedience, chastity, filial piety, and justice) are usually harmful for those who possess them […] But your neighbor praises your virtue precisely on this account.

Thus far, we have generally been assuming that benefits to the collective are worth the sacrifice of the individual’s interests. There is no doubt that in many cases this is true. Humans are a hyper-social, hyper-cooperative species. We would not be here if we weren’t often willing to forego individual benefit on behalf of the collectives to which we belong, especially since those sacrifices often redound to our benefit indirectly over time as our groups reap the rewards of cooperation in non-zero-sum interactions. But, in a world of relative abundance, we must also ask how much is too much, especially when the same types of individuals are asked, over and over again, to forego a benefit for the collective good. In *The Virtues*, Geach (1977) suggests that “Men need virtues as bees need stings.” The resonance and discord with Mandeville’s *Fable of the Bees* here is remarkable. Individual bees need their stings only in a very indirect, collective way: for female honeybees, to sting is often to die. Geach would have been more accurate to say that collectives need mandevillian virtues in their members as honeybee hives need their females to have stings.

In contemporary philosophy, the idea of type-relative virtues is almost absent or at least controversial (e.g., responses to what seems like gender essentialism in Gilligan, 1977). As O’Connor (2019) has persuasively argued, what makes type-based divisions of labor so tenacious is that they often *do* lead to collective benefits even as they introduce and entrench inequalities over the course of lifetimes and generations. If the group did not benefit from such type-based division of labor, it would wither. A question thus naturally arises: to what extent are we willing to tolerate persistent, type-based social inequalities in order to reap the rewards of the division of moral and cognitive labor? A utopian response would be to insist that alternative divisions of labor not based on gender, race, ethnicity, class, caste, and religion are possible and desirable. In light of O’Connor’s arguments, we do not find this particularly plausible. Another response would be to insist on turn-taking (e.g., I’m forgiving this week while you’re vindictive, then you’re forgiving next week while I’m vindictive) that would ensure that disadvantaged demographics were not always expected to adopt the mandevillian traits that benefit their group at their own expense. Unfortunately, turn-taking is not consistent with *dispositional* diversity as we have discussed it, since dispositions are not easily adopted or abandoned. A third response, which we tentatively endorse and which is consistent with O’Connor’s conclusions, is that there need to be systematic efforts—both at the individual and the group level—to recognize and redress the persistent inequalities that likely, if not inevitably, arise from the division of labor, including the division of moral and intellectual labor. These could come in the form of redistributive programs addressing income, wealth, recognition, emotional labor, and other goods. In this paper, we do not have space to propose detailed policies, but we would be remiss if we did not at least raise this issue.

## Conclusion

The traditional virtue-theoretic approach of improving our moral and epistemic conduct centers around the cultivation of stable virtues through education and individual training. There are two problems with this agent-centered approach. First, on an Aristotelian conception of virtue, it is exceedingly difficult to carry out this program successfully, since there are many more ways of being vicious than there are of achieving the virtuous mean, and the mean is harder to achieve than many correlative vices. Second, even if this strategy proved successful, it would compromise the dispositional diversity that can yield significant moral and epistemic benefits at the level of collectives. For this reason, many of the traits that have traditionally been considered virtues (e.g., mercy, apt deference, open-mindedness) function as mandevillian vices in the social contexts where most of our behavior and thinking takes place. However, expecting individuals to be sensitive and responsive to the social conditions that call for mandevillian virtues, such as vindictiveness and closed-mindedness, is also impractical. Rather than hoping to train individuals to adapt to their moral and epistemic environments, we are better off designing environments that advantageously harness our pre-existing dispositions (Alfano forthcoming; Bland, 2024; Levy, 2022). We already do this, using incentive structures and role-assignments to effectively distribute the moral and epistemic labor required for collective success (Astola, 2021), but there’s plenty of room for improvement. Part of this improvement must involve avoiding and remedying historical injustices in the division of labor.

## Footnotes

1 One of the few ways in which it can go awry relates to the knowledge norm of action (Fantl & McGrath 2009): a more capable individual is more likely to achieve knowledge or mistakenly believe that they know, which may lead them to take risks that a less capable individual might shy away from. This is not to say that stupid people don’t take stupid risks. They do. But smart people tend towards their own distinctive types of risky decisions as well, as anyone who has observed techbro solutionism can attest.

2 This phenomenon is nicely illustrated in this demonstration: url =  < https://ncase.me/trust/ > , accessed 26 June 2023.

3 An alternative, “forgetful” strategy leads agents to ignore rather than forgive past defections (Milinsky & Wedekind, 1998), which can have much the same effect of promoting cooperation. Such forgetfulness is arguably a mandevillian virtue: it’s not epistemically virtuous to be forgetful, but when people are forgetful in certain ways and contexts, they end up cooperating more effectively than their memorious counterparts.

4 We should be careful, however, not to assume that this taste for originality is uniform across populations. It may differ, for example, in WEIRD and non-WEIRD populations (Henrich, Heine & Norenzayan 2010).

5 In fact, Einstein’s intellectual independence served him poorly later in his life when he resisted the most plausible interpretations of quantum mechanics.

6 This is not to say that it completely succeeds in doing so. In fact, it seems that juries are less accurate than we want or expect them to be. Nevertheless, this is often a failure to live up to the ideal prescribed by the judicial system rather than an indictment of the ideal (Arkes & Mellers 2002).
